# Secondary indoor air pollution and passive smoking associated with cannabis smoking using electric cigarette device–demonstrative *in silico* study

**DOI:** 10.1371/journal.pcbi.1009004

**Published:** 2021-05-13

**Authors:** Kazuki Kuga, Kazuhide Ito, Wenhao Chen, Ping Wang, Jeff Fowles, Kazukiyo Kumagai

**Affiliations:** 1 Faculty of Engineering Sciences, Kyushu University, Kasuga-koen, Kasuga, Fukuoka, Japan; 2 Indoor Air Quality Program, Environmental Health Laboratory, California Department of Public Health, Richmond, California, United States of America; RMIT University, AUSTRALIA

## Abstract

With electronic (e)-liquids containing cannabis components easily available, many anecdotal examples of cannabis vaping using electronic cigarette devices have been reported. For electronic cigarette cannabis vaping, there are potential risks of secondary indoor air pollution from vapers. However, quantitative and accurate prediction of the inhalation and dermal exposure of a passive smoker in the same room is difficult to achieve due to the ethical constraints on subject experiments. The numerical method, i.e., *in silico* method, is a powerful tool to complement these experiments with real humans. In this study, we adopted a computer-simulated person that has been validated from multiple perspectives for prediction accuracy. We then conducted an *in silico* study to elucidate secondary indoor air pollution and passive smoking associated with cannabis vaping using an electronic cigarette device in an indoor environment. The aerosols exhaled by a cannabis vaper were confirmed to be a secondary emission source in an indoor environment; non-smokers were exposed to these aerosols via respiratory and dermal pathways. Tetrahydrocannabinol was used as a model chemical compound for the exposure study. Its uptake by the non-smoker through inhalation and dermal exposure under a worst-case scenario was estimated to be 5.9% and 2.6% of the exhaled quantity from an e-cigarette cannabis user, respectively.

## Author summary

How can we best mitigate unintended passive smoking in an indoor environment? As marijuana tends to be legalized in more countries, there is an increasing number of cases of vaping cannabis using e-cigarette devices. E-cigarette vaping is presumed to cause relatively low levels of indoor air pollution due to the absence of a direct combustion process. In this study, we developed a numerical simulation model to quantitatively predict the impact of first- and second-hand cannabis vaping in an indoor environment. The study was conducted in response to vulnerable residents who are concerned regarding the deterioration of indoor air quality and informs policymakers of the potential risk of second-hand cannabis vaping exposure.

## Introduction

Cannabis is the most used and abused illicit drug in the world: there were an estimated 188 million users of marijuana in 2017, corresponding to 3.8% of the global population aged 15–64 [1]. Heavy use of cannabis in adolescence is associated with cognitive impairments, such as a decline in learning efficiency and memory [2]. Furthermore, cannabis use can also lead to an addiction to cannabis, alongside the development of depression and psychosis [3–5]. Generally, the most preferred method of cannabis consumption for both medicinal and recreational users is smoking [2]. With a rapid increase in the use of electronic cigarettes (e-cigarette) in recent years, cannabis vaping is an additional concern. Typically, e-cigarette devices are used as alternatives to traditional cigarette smoking by vaporizing e-liquid which mainly contains propylene glycol, glycerol, nicotine, and flavorings [6]. However, these devices have been modified to allow cannabis use.

People most often use cannabis indoors [7,8], where it is associated with high concentrations of contaminants [9] that can be inhaled by other nonsmokers. Hence, the nonsmokers are passively exposed to secondhand cannabis smoke, given the current prevalence and patterns of cannabis use. The secondhand smoke of a conventional cigarette is the smoke exhaled by a smoker or side stream smoke generated from combusted products. However, during e-cigarette use, only exhaled smoke is produced, due to the lack of a combustion process. Therefore, the exhalation rate of a cannabis vaper is a dominant factor contributing to secondhand exposure in an indoor environment, especially in a low ventilated room. However, few studies have focused on the quantity of inhaled cannabis that is exhaled indoors during e-cigarette vaping [10,11].

In addition to the exhalation rate of cannabis vaping, how the exhaled cannabis vapor is transported to the breathing zone of bystanders indoors and surrounds them through the air is a significant concern. This is strongly dependent on the ventilation system, positions of the smokers, and the exhalation profile. Cannabis absorption via the skin surface may also be a significant exposure pathway, which remains unclear.

Regarding inhalation and dermal exposure to chemical compounds, experimental studies (i.e., in vivo and in vitro studies), involving human volunteers and other surrogate animals have been conducted [12–16]. These experimental approaches are restricted by ethical challenges. From this perspective, a computer simulation model (i.e. *in silico* model) is an alternative and complementary approach that may strongly aid the understanding of the contaminant transport mechanisms in the respiratory system and the absorption of contaminants onto the skin. To address the inhalation and dermal exposure risk, computational fluid dynamics (CFD) and physiologically based pharmacokinetic (PBPK) models have been applied to realistic respiratory tract models and a computational simulated person (CSP). Recently, Kuga et al. [17] successfully developed a numerical prediction method for inhalation and dermal exposure to contaminants generated from e-cigarettes based on the CFD technique and the PBPK model. They focused on representative volatile organic compounds (VOCs): formaldehyde, acetaldehyde, acrolein, benzene, toluene, glycerol, and nicotine, as target contaminants generated from e-cigarette use. It was found that the contribution rate depends on the type of chemical compound. For this study, we are interested in whether the contribution rates of cannabis generated from vaping are similar to those of other chemical compounds generated from nicotine-based e-cigarette use. Although recent study of Zhao et al [18] developed a computational fluid-particle dynamics (CFPD) plus pharmacokinetics (PK) model to quantify the localized vapor and particle uptake rates of cannabis and the cannabis concentrations in plasma using two human upper airway models, they did not focus on the exhalation and second-hand exposure including inhalation and dermal absorption by the passive smoker.

This study focuses on a qualitative and quantitative assessment of inhalation dosimetry of firsthand cannabis vaping in a human respiratory tract. It also assesses the impact of second-hand exposure including inhalation and dermal absorption by the passive smoker due to the diffusion and dispersion of exhaled cannabis vapor in an indoor environment. We developed a comprehensive numerical model and CSP to investigate the potential effects of e-cannabis vaping on local tissue dosimetry and indoor air quality (IAQ). We also demonstrated a numerical analysis for first- and second-hand cannabis exposure in an indoor environment.

## Materials and methods

A risk assessment of continuous exposure to first- and second-hand cannabis was carried out using a quasi-coupling simulation method of CFD, CSPs, and a numerical respiratory tract model that was validated from multiple perspectives for prediction accuracy in our previous study [17]. For the inhalation exposure analysis of firsthand cannabis vaping, flow patterns, temperature, and cannabis concentration distributions in a realistic numerical respiratory tract model were calculated using the CFD technique. The numerical respiratory tract model was developed based on computer tomography (CT) data extracted from a healthy human male. The flow patterns and temperature distribution were validated by PIV measurement and comparing to the experimental study [19,20]. This model contains approximately 2×10^6^ polyhedral elements and very fine prism layers (< 0.1 mm prism mesh), in the near-wall region to satisfy y^+^ < 1 in all respiratory surfaces under peak air velocity. To reproduce the shape variation of the mouth opening during the inhalation and exhalation period, this model has two different types of mouth opening (see S1 Fig). Additionally, to investigate the effect of puffing profiles on the total respiratory uptake, we assumed three transient puffing profiles: (i) short puffing, (ii) long puffing, and (iii) post puffing, which is set at the mouth opening as an inflow boundary condition. The first two puff profiles were created based on the measurement of puff profiles in use of e-cigarettes by Vansickel et al [21]. The exhalation profile was described using a sinusoidal profile based on an expiratory time of 1.8 seconds [22] and inhalation volume. A measured human post-puff profile was also used [23]. The turbulent kinetic energy at the circular inlet was defined assuming a turbulent intensity of 10%. Furthermore, for energy transport within the respiratory tract, the vapor temperature of the inhaled vapor was assumed to be 45°C based on previous experimental results [24,25]. The respiratory surface was assumed to have a constant temperature of 36.4°C. Additionally, the boundary conditions for the respiratory surface consider the diffusive transport to respiratory tissues; namely, the mucus, epithelium, and sub-epithelium, and the AMTB (Air-Mucus-Tissue-Blood) model proposed by Tian and Longest [26–28] was applied to the respiratory surface. To analyze the absorption fluxes with numerical stability, we applied the double boundary film theory based on the partition coefficient and the flux conservation between the air and tissue phases as the Dirichlet boundary condition in the wall surface of the air and tissue zones. This study selected tetrahydrocannabinol (THC) as a target chemical component. The physical properties of THC for inhalation exposure analysis were estimated.

Concerning the exhalation and passive smoking of cannabis vapor released into indoor environments (secondhand cannabis smoking), we assumed a simple cubic room model (3m x 3m x 3m) with a displacement ventilation system, where there are two CSPs standing face to face with a distance of 1m (see S3 Fig). These CSPs were assumed an active smoker and passive smoker. In the assumed ventilation system, the fresh outdoor air is entering form in a small inlet opening with a velocity of 0.2 m/s. The supply air temperature was 22°C and the turbulent intensity was assumed at 10%. The ventilation rate was 0.018 m^3^/s, providing the ventilated room with a general air change rate of 0.6 h^-1^. The flow patterns and temperature distribution surrounding human bodies caused by thermos-physiological sensible heat generation were reproduced by using a simple algorism for the skin surface temperature [29,30]. A demonstrative scenario for exhalation and passive smoking was assumed: cannabis is released into the indoor environment through the e-cigarette cannabis user’s post puff, and then the passive smoker inhales the exhaled cannabis vapor via nasal breathing and also absorbs cannabis vapor through the dermal pathway. In terms of the nasal breathing, we applied the nasal breathing cycle model proposed by Gupta et al. [31]. Quasi-coupling with time-varying results of inhalation exposure analysis in the respiratory tract model reproduced the exhalation of cannabis vaping. Additionally, to simulate short-term dermal exposure to cannabis vapor released from exhalation, a specific dermal absorption model based on the transdermal model proposed by Morrison et al. [32] was applied to the skin surface of CSPs. The physical properties of THC for dermal exposure analysis were also estimated by several assumptions.

The detailed system of governing equations and boundary conditions are provided in the SI.

## Results and discussion

### Primary inhalation exposure of cannabis users in e-cigarette

**Fig 1:**
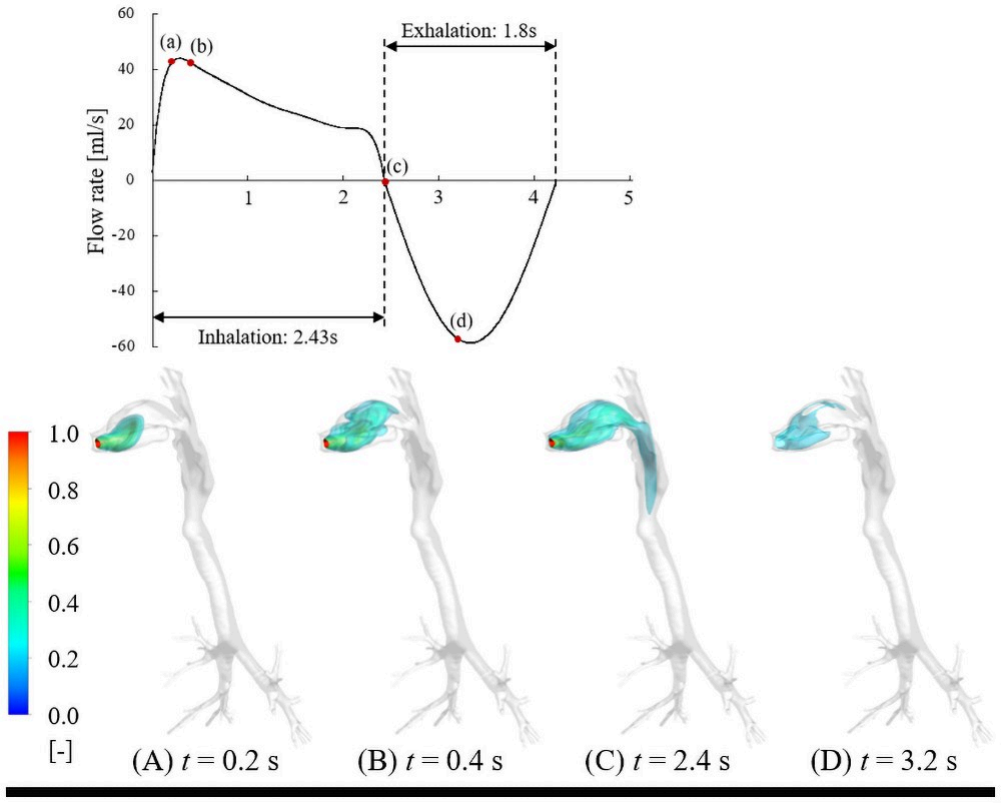
Time evolution of isosurfaces of non-dimensionalized THC concentration in respiratory tract under short puff.

**Fig 2:**
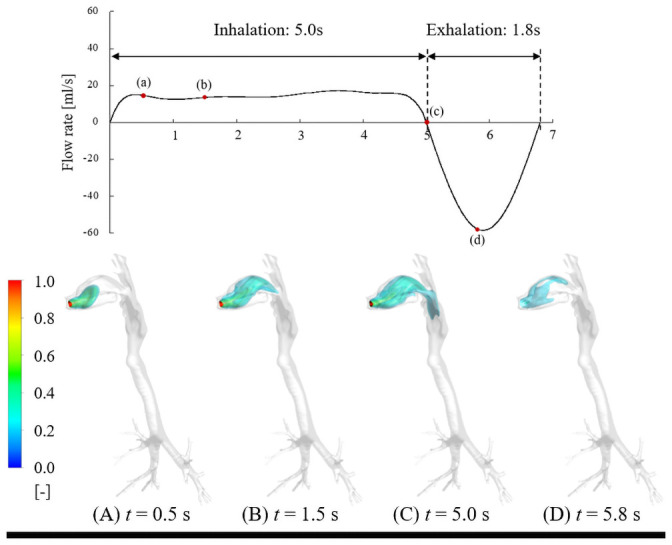
Time evolution of isosurfaces of firsthand THC concentration in respiratory tract under long puff.

**Fig 3:**
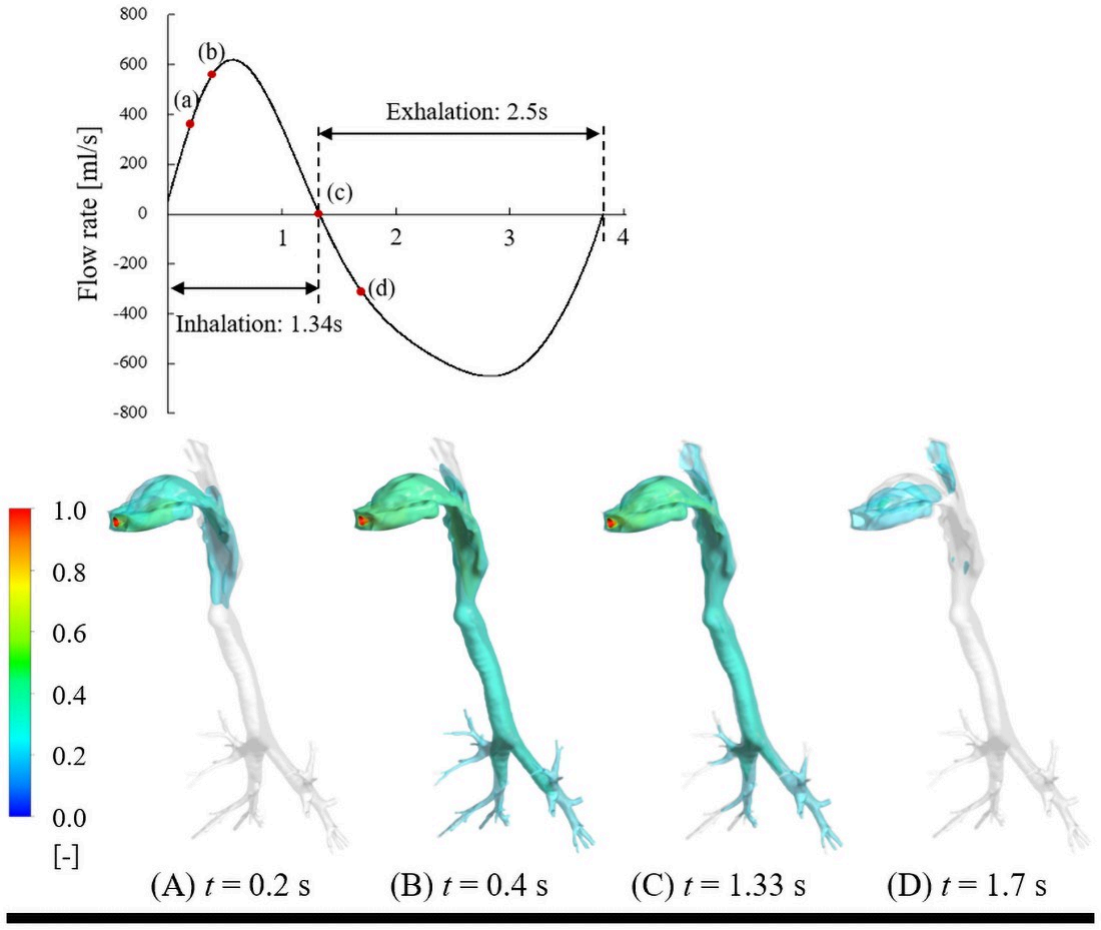
Time evolution of isosurfaces of firsthand THC concentration in respiratory tract under post puff.

The time evolution of isosurfaces of firsthand tetrahydrocannabinol (THC) concentrations in the respiratory tract under short puff (inhalation time: 2.43 s, exhalation time: 1.8 s), long puff (inhalation time: 5.0 s, exhalation time: 1.8 s), and post puff (inhalation time: 1.34 s, exhalation time: 2.5 s) are shown in Figs 1, 2 and 3, respectively. THC concentrations are normalized by the inhaled THC concentration. As shown in Figs 1, 2 and 3, the THC concentration distributions are significantly influenced by puffing profiles with different puffing flow rates and puffing durations.

For the short puff condition, the inlet jet led to a higher concentration of cannabis vapor near the tongue region and enhanced the absorption of cannabis vapor onto the respiratory surface of this region at *t* = 0.2 s (Fig 1A). Additionally, the impact of the inlet jet resulted in the mixing of a high concentration of cannabis vapor and the transportation of cannabis vapor to the upper palate at *t* = 0.4 s (Fig 1B). After this, the cannabis vapor was transported to the larynx and trachea through the oropharynx until the end of the inhalation period (*t* = 2.4 s, Fig 1D). In the exhalation period, almost all the cannabis vapor was released out of the respiratory tract from the mouth opening within approximately 0.9 s (*t* = 3.3 s, Fig 1D). The exhaled cannabis vapor passed through the upper palate because of the shape of the pharynx region. Therefore, the absorption of cannabis on the upper palate region was larger than the tongue region.

Although the THC concentration distribution under the long puff condition was similar to that of the short puff condition because the total puff volume was almost identical, the time to form a similar distribution of each state is different due to the different puff durations and peak flow rates (Fig 2). Additionally, the long puff condition led to less transportation to the larynx region than the short puff condition because of the absorption rate and low flow rate.

In contrast to the short and long puff conditions, the post puff condition with a high flow rate promoted the transportation of cannabis vapor to the lung region and reached the fourth generation of bronchiole tubes within a short time (approximately 0.4 s, Fig 3B). The remaining cannabis vapor was vigorously removed by exhalation.

Cannabis inhaled by vaping can be adsorbed by respiratory surfaces/tissues, exhaled to indoor air, remain in the respiratory tract, or be transported to the lungs as a gas. The contributions of THC: (a) absorption rate, (b) exhalation rate, and (c) rate of remaining respiratory tract or transport lungs, was calculated, with the total inhalation rate counted as 100%. The absorption rate was 84.1%, 85.0%, and 64.4% for short puff, long puff, and post puff, respectively. The absorption rate under the long puff condition with the long puff duration was higher than that of the other puff conditions with higher puff intensity. This result indicated that the puff duration was a dominant factor in total absorption. Additionally, 6.6% of inhaled cannabis vapor was exhaled to the indoor environment under both the short and long puff conditions. However, the exhalation rate under the post puff condition was 3.2%, which was relatively low compared to the short and long puff conditions because of the high rate of transport to the lungs of 32.4%. In the case of short and long puff conditions, the cannabis vapor was hardly transported to the lungs and a small portion of inhaled cannabis vapor remained in the respiratory tract without absorption and exhalation (9.3% for short puff, 8.4% for long puff). To the best of our knowledge regarding the numerical simulations of inhalation exposure, there is no study focusing on cannabis vaping. Our previous study focused on seven chemical compounds as representative VOCs generated from e-cigarettes. The calculated absorption rate was: formaldehyde (88 ~ 95%), acetaldehyde (32 ~ 81%), acrolein (23 ~ 72%), benzene (0.2 ~ 2.2%), toluene (0.3 ~ 3.1%), glycerol (76 ~ 90%), and nicotine (70 ~ 86%) [17]. The absorption rate of benzene and toluene was very low compared to the other chemical compounds because of their low solubility (low partition coefficient). Compared to our previous study, the absorption rate of THC in cannabis vaping was similar to that of nicotine. Conversely, the rate of THC remaining in the respiratory tract was higher than that of nicotine (1.6% for short puff, 1.4% for long puff). This result may be caused by a lower diffusion coefficient of THC. As a result, the exhalation rate of cannabis would be lower than that of nicotine, which would strongly affect secondhand exposure.

According to a report by Lee and Hancox [33], cannabis smokers tend to take much deeper breaths and employ breath-holding techniques to increase the absorption of THC as bioavailability ranges from 18 to 50%, depending on the volume of air inhaled, the depth of inhalation, and the duration of retention of smoke in the alveoli. Considering the continuous desorption of THC from the respiratory surface after puffing, it is reasonable that the absorption rate in our simulation was higher than the bioavailability ranges.

### Secondhand exposure to exhaled cannabis vapor

**Fig 4:**
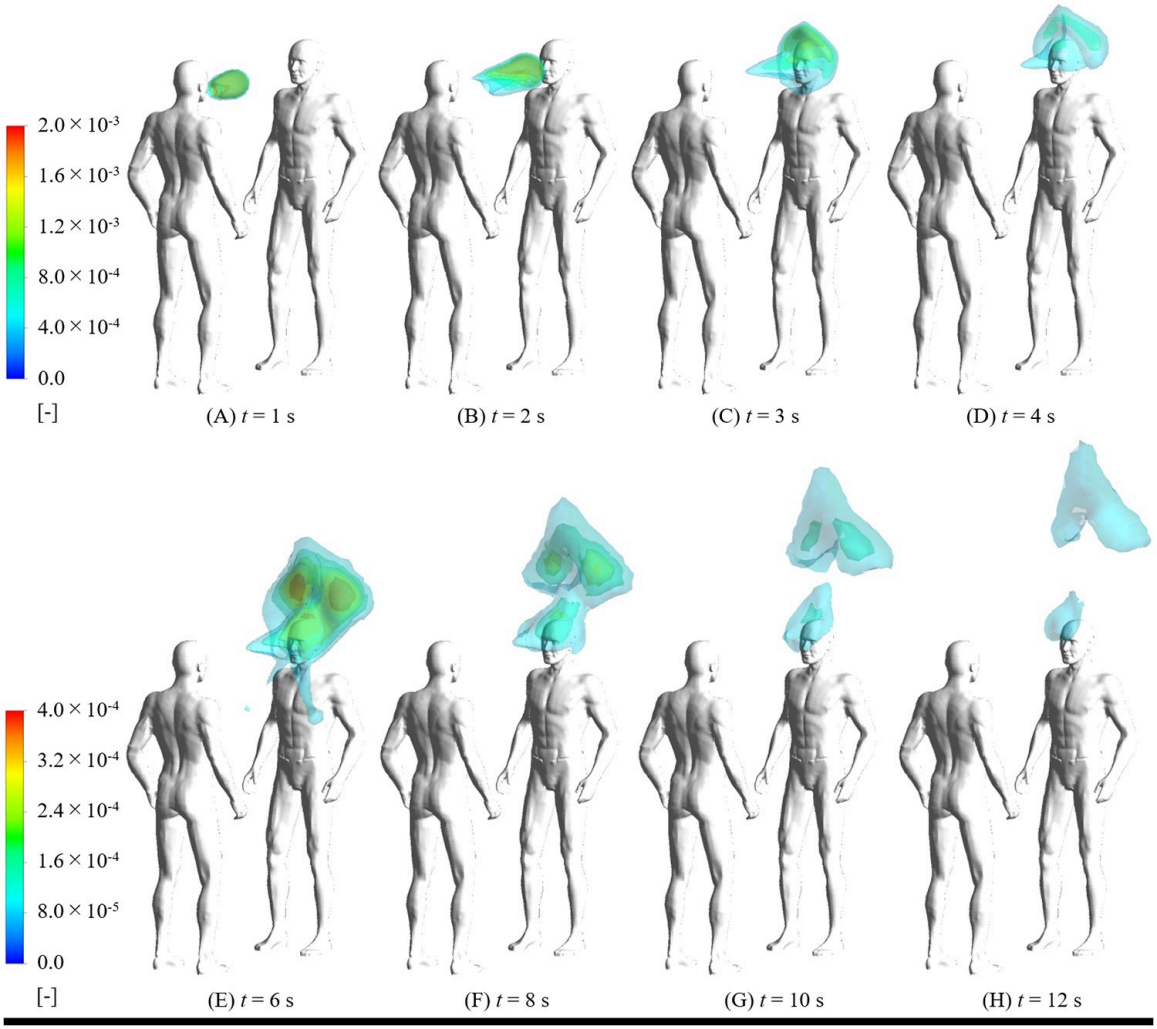
Time evolution of isosurfaces of THC concentration exhaled from cannabis user in e-cigarette under post puff profile and exposure to passive smoker.

The time evolution of isosurfaces of THC concentration exhaled from the e-cigarette cannabis user under a post puff profile and exposure to a passive smoker is shown in Fig 4. The cannabis user exhaled cannabis vapor that remained in the respiratory tract to the indoor environment. As shown in Fig 4A and 4B, the exhaled cannabis vapor was transported straight to the front of the passive smoker’s face by exhalation in approximately 2.0 s. After this, the passive smoker inhaled 5.9% of exhaled cannabis vapor by nasal breathing three times, and absorbed 2.6% of exhaled cannabis vapor through the skin surface of the face until *t* = 12 s. Under the model ventilation and room volume conditions (details are described in the supplemental information), we can assume the exhaled THC is similar to a pulse generation phenomenon when compared to a nominal time scale. Hence, the exhaled THC concentration was diluted instantaneously in the indoor environment. Additionally, most of the cannabis vapor suspended in the room without inhalation or dermal absorption rose to the ceiling and may be exhausted outdoors. For short and long puffing behavior, the exhaled cannabis vapor did not arrive at the passive smoker and rose to the ceiling along the thermal plume generated from the e-cigarette cannabis user because of the low flow rate during exhalation. Thus, if the e-cigarette cannabis user softly exhaled vapors, the health effect of secondhand cannabis exposure would be almost zero.

**Fig 5:**
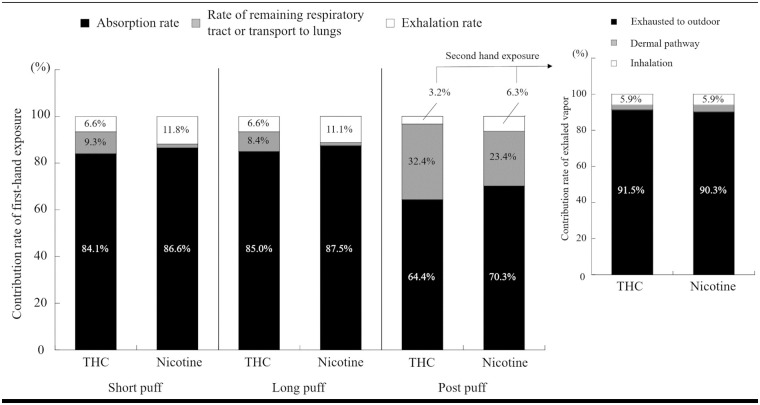
Contribution rate of THC and nicotine generated from one puff of e-cigarette device from firsthand to secondhand exposure under post puff scenario.

Fig 5 shows the contribution rate of THC and nicotine generated from one puff of an e-cigarette device from firsthand to secondhand exposure under the post puff scenario. The dermal absorption and inhalation portions are 2.6% and 5.9% of exhaled THC, respectively. Considering that exhalation accounts for 3.2% of the total inhaled quantity of THC, the dermal absorption and inhalation portions are 0.08% and 0.19% of the total inhaled quantity of THC, respectively. If we assume that e-liquid contains 0.5 g of THC oil which is at 80% concentration, and an e-cigarette user can take 100 puffs from a 0.5 g THC oil, the inhalation THC dosage is 4 mg per puff [34]. Combined with our simulation results, 2.6 mg THC would be absorbed into the respiratory surface for one post puff, and 1.3 mg THC would be transported to the lungs or remain in the respiratory tract without absorption and exhalation. Additionally, the exhaled THC would be 0.1 mg per puff, which affects indoor air quality and secondhand exposure. According to Behar et al. [35], e-cigarette users generally take more than 20 puffs for a 10-min session of e-cigarette use. Therefore, at least 2 mg THC would be emitted to indoor air from an e-cigarette cannabis user for 10-min. Furthermore, in the worst-case scenario of secondhand exposure where the e-cigarette cannabis user exhaled THC with a high flow rate to a nearby passive smoker, the passive smoker would inhale 7.6 μg THC and absorb 3.2 μg THC for one e-cigarette user’s puff. Conversely, when the e-cigarette cannabis user exhaled vapors with a low flow rate such as short and long puff behavior, the inhalation and dermal dosage was not detected in short-term exposure. Thus, the differences in puff profiles and behaviors were confirmed to have a potential impact on not only firsthand exposure to inhaled cannabis vapor but also secondhand exposure to exhaled cannabis vapor.

In summary, we used the previously developed numerical analyses methods using computer simulated persons with respiratory tract and indoor CFD techniques to explore the contribution rate of first- and secondhand exposures to THC generated from e-cigarette device. For the results of these numerical analyses, the contribution rate of THC was similar to that of nicotine in both of first- and secondhand exposure. Additionally, the contribution rates of exhaled vapor strongly depended on the puffing behavior. This implies that softly exhaling vapor is recommended for minimal impact to the non-smoker/bystander. This study focused on the short-term exposure and reproduce only single e-cigarette puffing. To discuss the long-term exposure risk, reproducing multiple puffs and development of numerical model corresponding to the long-term exposure will be required in future.

## Supporting information

S1 Text Online supplemental information. (DOCX)

S1 Fig Numerical respiratory tract model. (TIF)

S2 Fig System of one-dimensional transient diffusion equations for THC absorption into respiratory tissues. (TIF)

S3 Fig Analytical domain for the exhaled cannabis dispersion and passive smoking. (TIF)

S4 Fig System of one-dimensional transient diffusion equations for THC dermal absorption. (TIF)
