# Supplementary material for: Secondary indoor air pollution and passive smoking associated with cannabis smoking using electric cigarette device–demonstrative in silico study
Source: PLoS Comput Biol. 2021 May 13;17(5):e1009004. doi: 10.1371/journal.pcbi.1009004 (PMC8148323; doi:10.1371/journal.pcbi.1009004)
Supplement: S1 Text — (DOCX) [file pcbi.1009004.s001.docx]

**Supporting Information**

Secondary indoor air pollution and passive smoking associated with cannabis smoking using electric cigarette devise – Demonstrative in silico study

Kazuki Kuga, Kazuhide Ito, Wenhao Chen, Ping Wang, Jeff Fowles, Kazukiyo Kumagai

Kazuki Kuga

Email: [kuga@kyudai.jp](mailto:kuga@kyudai.jp)

**Materials and Methods**

**S1. Inhalation exposure analysis for cannabis vaping**

*Numerical respiratory tract model*

The numerical respiratory tract model used in this study includes the oral cavity, pharynx, larynx, trachea and bronchial tubes reproduced around the fourth bifurcations with 38 outlet openings. We had confirmed that flow patterns in the respiratory tract did not depend on the existence of the nasal cavity region prior to inhalation exposure analysis using the respiratory tract excluding nasal cavity region [1,2]. The airway model had already been developed based on computer tomography (CT) data extracted from a healthy human male. This model was reported in terms of airflow and heat/mass transport along with requisite validation [3,4]. This model included approximately 2×10^6^ polyhedral elements and very fine prism layers (< 0.1 mm prism mesh), in the near-wall region to satisfy y^+^ < 1 in all respiratory surfaces under peak air velocity. To simulate the inhalation of the e-cigarette vapor in the respiratory tract model, a circular inlet opening with a diameter of 8.0 mm was set to simulate a stream of vapor coming from the mouth directly into the pharynx region. An elliptical outflow opening with an area of 1.2 cm^2^ [5] was established to reproduce the shape of mouth opening during the exhalation period.

*Governing Equations*

Flow patterns, temperature, and THC concentration distributions under e-cigarette puffing conditions were calculated using CFD analysis. Assuming inhaled cannabis vapor is diluted in the air, the air-vapor mixture can be modeled as a single continuous phase. The governing equations of the air-vapor mixture were:

*Continuity Equation*

$\frac{\partial\overline{U_{i}}}{\partial x_{i}}=0$, (S-1)

in which $\overline{U_{i}}$ represents the ensemble mean i-th component of the continuous phase velocity.

*Navier-Stokes (N-S) Equation*

$\frac{\partial\overline{U_{i}}}{\partial t}+\frac{\partial\overline{U_{j}}\overline{U_{i}}}{\partial x_{i}}=-\frac{1}{\rho}\frac{\partial p}{\partial x_{i}}+\frac{\partial}{\partial x_{j}}\left\{\nu\left(\frac{\partial\overline{U_{i}}}{\partial x_{j}}+\frac{\partial\overline{U_{j}}}{\partial x_{i}}\right)-\overline{u_{i}^{'}u_{j}^{'}}\right\}-\beta(\overline{T}-\overline{T_{0}})g_{i}$, (S-2)

where *ρ* and *ν* are the air vapor mixture density and viscosity, respectively. $\overline{u_{i}^{'}u_{j}^{'}}$ is the Reynolds shear stress.

The low Reynolds (Re) number type k-ε model (Abe–Kondoh–Nagano model) [6,7] was adapted to simulate flow patterns in the respiratory tract model because it provides an accurate and numerically efficient solution for transitional and turbulent flows. Specifically, reasonable prediction accuracy of low Re number type k-ε model for air flow distribution analysis in the respiratory tract was validated using particle image velocimetry (PIV) [3].

*Convection-Diffusion equation for cannabis transportation*

The contaminants transport equation for turbulent flow can be expressed in terms of contaminant concentration as:

$\frac{\partial\overline{C_{a}}}{\partial t}+\frac{\partial\overline{U_{i}}\overline{C_{a}}}{\partial x_{i}}=\frac{\partial}{\partial x_{i}}\left\{\left(D_{a}+\frac{\nu_{t}}{\sigma}\right)\frac{\partial\overline{C_{a}}}{\partial x_{i}}\right\}$, (S-3)

where $\overline{C_{a}}$ is the ensemble mean contaminant concentration; *D_a_* is the diffusion coefficient of contaminant in air; *ν_t_* is the turbulent viscosity; vectors Ui and xi are the fluid velocity and position, respectively; and *σ* is the turbulent Schmidt number.

*Inflow and outflow boundary conditions*

The velocity profiles were directly set in the mouth opening as inflow and outflow boundary conditions. In this study, to investigate the effect of puffing behavior in terms of total respiratory uptake, three types of puffing profiles were prepared: (i) short puff, (ii) long puff, and (iii) post puff, shown in Fig. S2. The first two puffing profiles were created based on the measurements of puff profile during usage of a prototype e-cigarette in exclusive cigarette smokers and e-cigarette users by Vansickel et al [8]. The exhalation duration for the two profiles was set as 1.8 s because no difference was observed in exhalation duration between traditional cigarette smokers and e-cigarette users [9]. The exhalation profile was described using a sinusoidal profile based on exhalation duration and inhaled volume. A measured human post-puff profile was also used [10]. In this study, three types of transient puffing profiles were set in the mouth opening as the inflow/outflow boundary condition. The turbulent kinetic energy at the circular inlet was prescribed by assuming 10% turbulence intensity. In addition, for energy transport in the respiratory tract, the inhaled e-cigarette vapor temperature was assumed as 45 °C based on previous experimental results [11,12]. The respiratory surfaces were assumed to be at a constant temperature of 36.4 °C.

*Wall boundary condition for THC absorption onto respiratory tissues*

The absorption rate of THC into the respiratory tissues was determined by simultaneously solving the system of one-dimensional transient diffusion equations illustrated in Fig. S2. This system is based on the PBPK model proposed by Tian and Longest [13-15].

Parameters *H_m_*, *H_t_*, and *H_b_* are the thicknesses of the mucus, epithelium, and sub-epithelium in a specific region of the respiratory tract, respectively. *C_m_*(*y*, *t*), *C_t_*(*y*, *t*), and *C_b_*(*y*, *t*) are the THC concentrations in the mucus, epithelium, and sub-epithelium with units of μg/m^3^. These concentrations are spatially and time dependent in each material. *D_m_*, *D_t_*, and *D_b_* are the THC diffusivities in mucus, epithelium, and sub-epithelium with unit of m^2^/s. *Q_b_* is the blood flow rate in the sub-epithelium with unit of ml/s. *V_b_* is the volume of sub-epithelium with unit of ml. Initially, zero concentration is assumed in the mucus, epithelium, and sub-epithelium. At each interface, the relationship of partition coefficient (i.e., Henry constant of linear type adsorption isotherm) was satisfied. In addition, flux was conserved. Here, *P_ma_*, *P_tm_* and *P_bt_* are the mucus-air partition coefficient, the tissue-mucus partition coefficient, and the blood-tissue partition coefficient, respectively.

To analyze the absorption fluxes with numerical stability, we applied the double boundary film theory based on the partition coefficient and the flux conservation between the air and tissue phases as the Dirichlet boundary condition in the wall surface of the air and tissue zones. For example, the wall surface concentrations of the air and tissue phases can be represented by equations (S-4) and (S-5), respectively:

$C_{a,0}=\left(C_{a,1}+\frac{D_{m}}{D_{a}}\frac{\Delta x_{a}}{\Delta x_{m}}C_{m,1}\right)/\left(1+\frac{D_{m}}{D_{a}}\frac{\Delta x_{a}}{\Delta x_{m}}P_{ma}\right)$ (S-4)

$C_{m,0}=\left(C_{m,1}+\frac{D_{a}}{D_{m}}\frac{\Delta x_{m}}{\Delta x_{a}}C_{a,1}\right)/\left(1+\frac{D_{a}}{D_{m}}\frac{\Delta x_{m}}{\Delta x_{a}}\frac{1}{P_{ma}}\right)$ (S-5)

where *C_a,0_* and *C_m,0_* are wall surface THC concentrations of the air and mucus phases, and *C_a,1_* and *C_m,1_* are defined as the concentrations of the closest elements to the wall surface of the air and tissue zones. The wall surface THC concentrations are calculated using a sufficient number of iterations.

*Estimation of the physical properties of THC for inhalation exposure analysis*

Few studies have reported the physical properties of THC. Therefore, we estimated the physical properties of THC using some assumptions and empirical equations. The diffusivity of THC in air is estimated by empirical equation proposed by Ha and Kwon [16]:

$D_{a}=\frac{0.000155}{MW^{0.65}}$, (S-6)

where *MW* is the molecular weight of THC (314 g/mol).

The physical properties of the mucus including the diffusivity and partition coefficient was assumed equal to those found in water since mucus contains ~95 % water [17]. The diffusivities of THC in water were estimated by the Stokes-Einstein equation:

$D=\frac{KT}{6\pi\eta r_{s}}$, (S-7)

where *K* is the Boltzmann constant; *T* is temperature; *η* is the viscosity of water; and *r_s_* is the solute radius (Å). We hypothetically set the viscosity of water as 7.0×10^-4^ Pa s and approximated the solute radius by the empirical equation using the molecular weight (*MW*) [18]:

$4/3r_{s}^{3}=0.2894MW$, (S-8)

The diffusivity in the sub-epithelial tissue layer (blood phase) is also calculated by the Stokes–Einstein equation, assuming the viscosity of blood as 4.0×10^-3^ Pa s. The diffusivity in the epithelial tissue layer is determined as one-third that of mucus [19]. These estimation leads to *D_a_* = 0.037 cm^2^/s, *D_m_* = 7.63×10^-6^ cm^2^/s, *D_t_* = 2.54×10^-6^ cm^2^/s, and *D_b_* = 1.34×10^-6^ cm^2^/s, respectively.

In addition, the partition coefficient in each interface of the respiratory tissue layers is estimated based on various sources and assumptions [20]. The tissue-mucus partition coefficient (*P_tm_*) was quantified using the following equation [21]:

$P_{tm}=\frac{1}{P_{bt}}\frac{P_{ba}}{P_{ma}}$, (S-9)

where *P_bt_* is the blood-tissue partition coefficient, which is assumed to equal one [22]; and *P_ba_* is the blood-air partition coefficient, predicted by the following equation given by Buist et al. [23]

$\log P_{ba}=6.96-1.04\log VP-0.533\log P_{ow}-0.00495MW$ (S-10)

where *P_ow_* is the octanol-water partition coefficient (log *P_ow_* = 6.99 [24]); *VP* is the vapor pressure of THC (2.75 × 10^-5^ Pa at 25℃ predicted by Lovestead and Bruno [25]).

These estimation leads to *P_ma_* = 1.02 × 10^5^, *P_tm_* = 28, *P_bt_* = 1, and *P_ba_* = 2.84 × 10^6^, respectively.

# **S2. Dispersion of exhaled cannabis vapor and passive smoking**

*Computer simulated person and room model*

Computer simulated person (CSP) has been developed to reproduce the detailed shape of a human body and respiration phenomena for CFD analysis [26-30]. In this transient analysis for exhaled cannabis vapor dispersion and passive smoking of the resident, we used two types of CSPs standing face to face with a distance of 1m in a simple cubic room model (3m x 3m x 3m) with a displacement ventilation system. One of the CSPs is the e-cigarette cannabis user (i.e., an active smoker), and the other is a resident (i.e., a passive smoker). The analytical domain for the exhaled cannabis vapor dispersion and passive smoking is illustrated in Fig. S3.

*Boundary conditions for room model*

In the assumed ventilation system, the fresh outdoor air is entering form in a small inlet opening with a velocity of 0.2 m/s. The supply air temperature was 22 ℃ and the turbulent intensity *I*, was assumed at 10 %. The ventilation rate was 0.018 m^3^/s, providing the ventilated room with a general air change rate of 0.6 h^-1^. The exhaust outlet opening was located at the ceiling level. The walls of room model are assumed adiabatic condition.

*Inflow boundary conditions for CSPs’ breathing*

The e-cigarette user released the cannabis vapor into the indoor environment via exhalation. The flow boundary condition of the mouth opening was quasi-coupled with time-dependent results (velocity, turbulence, temperature, and contaminant concentrations) of inhalation exposure analysis in the respiratory tract model. In this study, the post-puff condition focused on second-hand exposure analysis. This was because the exhaled contaminants in the post-puff can be easily transported to the passive smoker due to the higher flow rate compared with short and long-puff conditions. Therefore, the post-puff condition leads to a high exposure risk and the worst case scenario for the passive smoker. On the other hand, the passive smoker inhales the exhaled cannabis vapor via nasal breathing and also absorbs it through the dermal pathway. In terms of the nasal breathing, we applied the nasal breathing cycle model proposed by Gupta et al. [5].

*Wall boundary condition for CSPs’ thermos-physiological sensible heat generation*

To reproduce the thermo-physiological sensible heat generation from human bodies, we applied a simple algorism for skin surface temperature, so-called Fanger’s thermo-regulation model [31,32]. Assuming that the flow patterns surrounding to CSPs are steady-state, the flow patterns and temperature distribution under steady-state condition are used as the initial condition for the analysis of exhaled cannabis dispersion and passive smoking (see Fig. S3).

*Wall boundary condition for dermal exposure to passive smoker*

The dermal absorption rate of THC is determined by simultaneously solving the system of one-dimensional transient diffusion equations illustrated in Fig. S4. This system is based on the PBPK model proposed by Morrison et al. [33].

*C_SSL_* and *C_SC_* are the THC concentrations in the skin surface lipid (SSL) and stratum corneum (SC) with unit of μg/m^3^, respectively. *D_SSL_* and *D_SC_* are diffusion coefficients in the SSL and SC with unit of m^2^/s, respectively. *H_SSL_* and *H_SC_* are the thickness of SSL and SC with unit of m, respectively. The skin tissue initial concentrations were assumed as zero. The local equilibrium was assumed at the air-SSL and SSL-SC interfaces, using the partition coefficient. The flux was conserved at each interface. Here, *P_SSL:a_* and *P_SC:SSL_* are the lipid-air partition and SC-lipid partition coefficients, respectively.

To analyze the absorption fluxes with numerical stability, we applied the similar Dirichlet boundary condition expressed as equations (S-4) and (S-5) to the skin surface of passive smoker.

*Estimation of physical properties of THC for dermal exposure analysis*

The diffusivity of THC in the SSL and SC (*D_lipid_*, *D_SC_*) and the partition coefficient in air-SSL interface and SSL-SC interface (*P_SSL:a_*, *P_SC:SSL_*) are estimated based on several assumptions. In the SSL, the diffusion coefficient was related to the solute radius as described in the literature [34,35]:

$D_{lipid}=\left\{\begin{matrix} 2\times10^{-9}\exp(-0.46r_{s}^{2}),\text{ }MW\leq380Da \\ 3\times10^{-13},\text{ }MW>380Da \end{matrix}\right.$ (S-11)

The effective diffusion coefficient in the SC was determined assuming that the corneocytes were impermeable and using the theoretical equation described by Kushner *et al* [36]*.*:

$D_{sc}=\frac{D_{lipid}}{\tau_{flux}\tau_{volume}}$ (S-12)

where *τ_flux_* and *τ_volume_* are tortuosity factors to account for parallel and branched transport or active in lipid region. *τ_flux_* and *τ_volume_* were calculated from the geometric parameters of SC:

$\tau_{volume}=\frac{Nh+(N-1)g+(N-1)d}{Nh+(N-1)g}$ (S-13)

$\tau_{flux}=\frac{Nh+(N-1)g+(N-1)\frac{\omega}{(1-\omega)^{2}}d}{Nh+(N-1)g}$, (S-14)

where *N*=12 is the number of corneocyte layers; *h*=0.88 (μm) is the thickness of the corneocyte; *g*=0.075 (μm) is the width of the lipid channel; *ω*=8 is the offset ratio; and *d*=40 (μm) is corneocyte width. These estimation leads to *D_lip_* = 9.28 × 10^-13^ cm^2^/s, *D_SC_* = 3.20 × 10^-15^ cm^2^/s, respectively.

It is typically difficult to directly measure the *P_SSL:a_*, as such, the *P_SSL:a_* was obtained by multiplying the water-air partition coefficient (*P_w:a_*) and the lipid-water partition coefficient (*P_lipid:w_*):

$P_{SSL:a}=P_{lipid:w}\times P_{w:a}$ (S-15)

The *P_SC:SSL_* was obtained by dividing the SC-water partition coefficient (*P_SC:w_*) in the *P_lipid:w_*:

$P_{SC:SSL}=\frac{P_{SC:w}}{P_{lipid:w}}$ (S-16)

The coefficients *P_lipid:w_* and *P_SC:w_*, were estimated assuming that the SC was partially hydrated (0.43 g water/1 g dry SC). Using the empirically derived equation described by Nitsche *et al* [37]*.*, we get:

$P_{lipid:w}=0.43P_{ow}^{0.81}$ (S-17)

$P_{SC:w}=0.040P_{ow}^{0.81}+4.06P_{ow}^{0.27}+0.359$ (S-18)

These estimation leads to *P_SSL:a_* = 2.01 × 10^10^, *P_SC:SSL_* = 0.09, respectively.


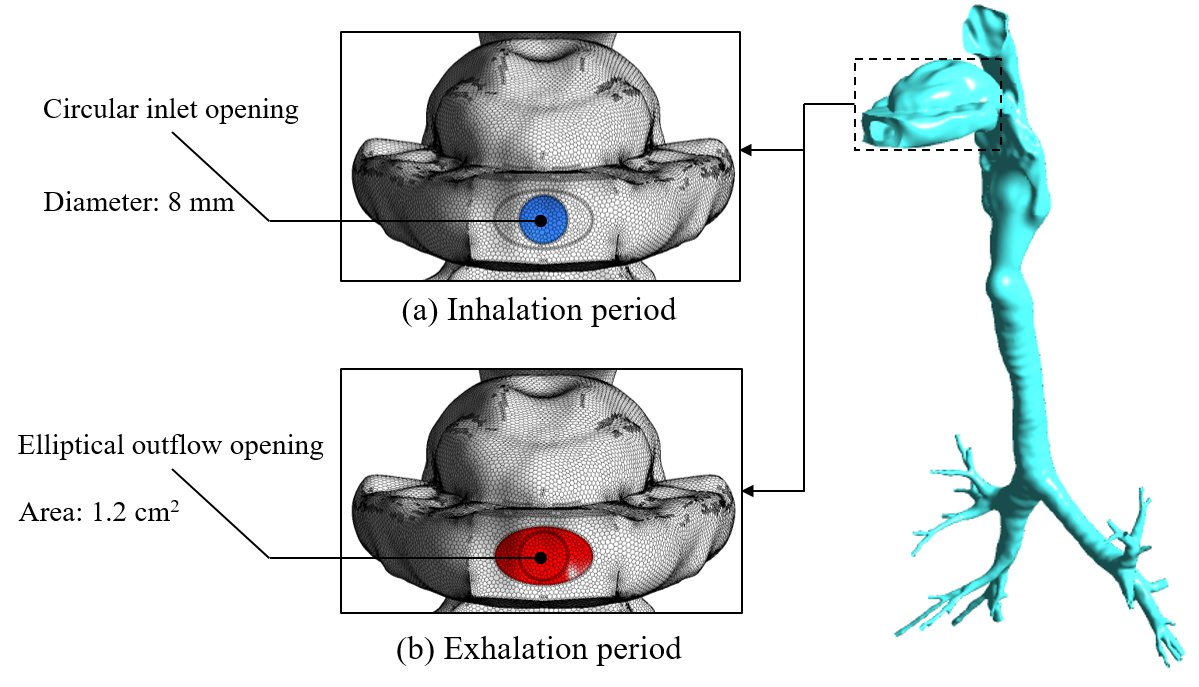


Fig. S1. Numerical respiratory tract model


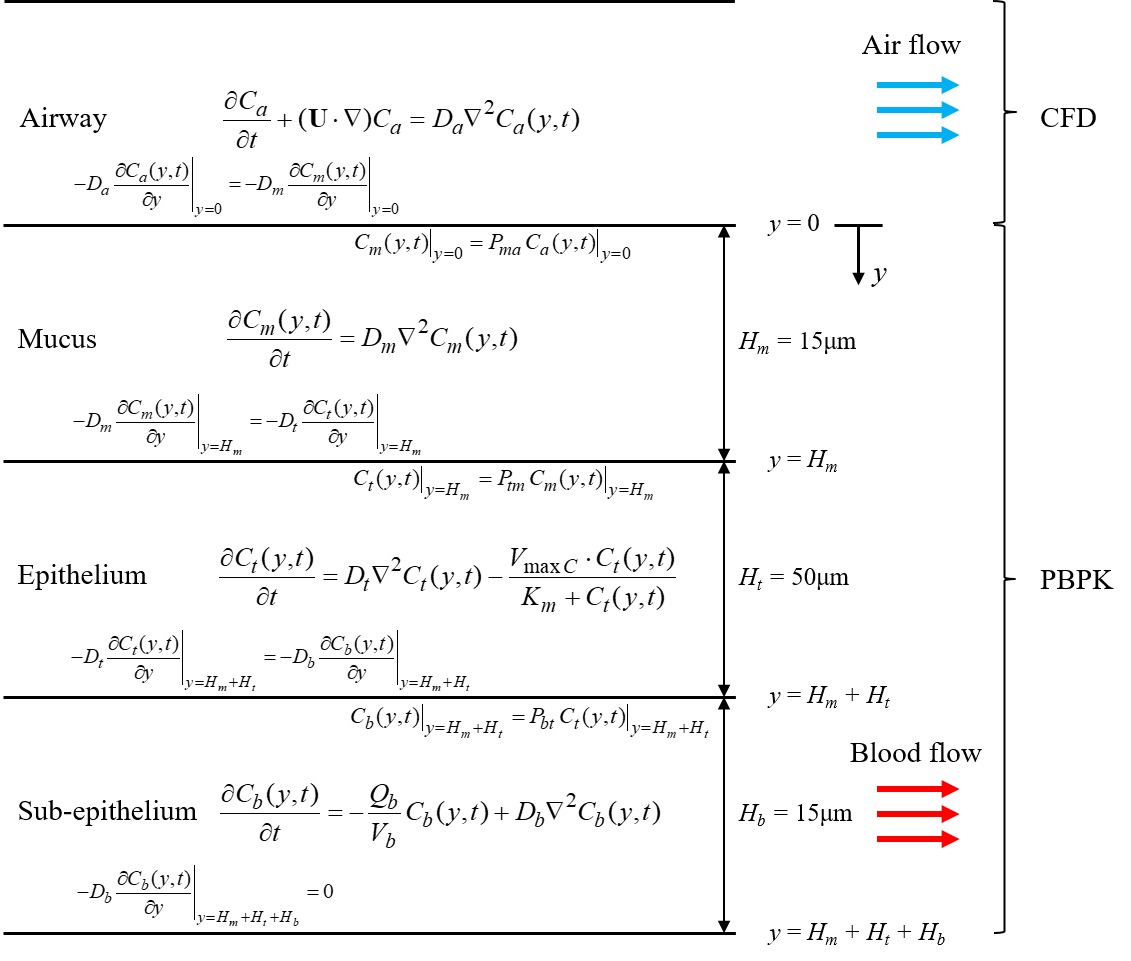


Fig. S2. System of one-dimensional transient diffusion equations for THC absorption into respiratory tissues


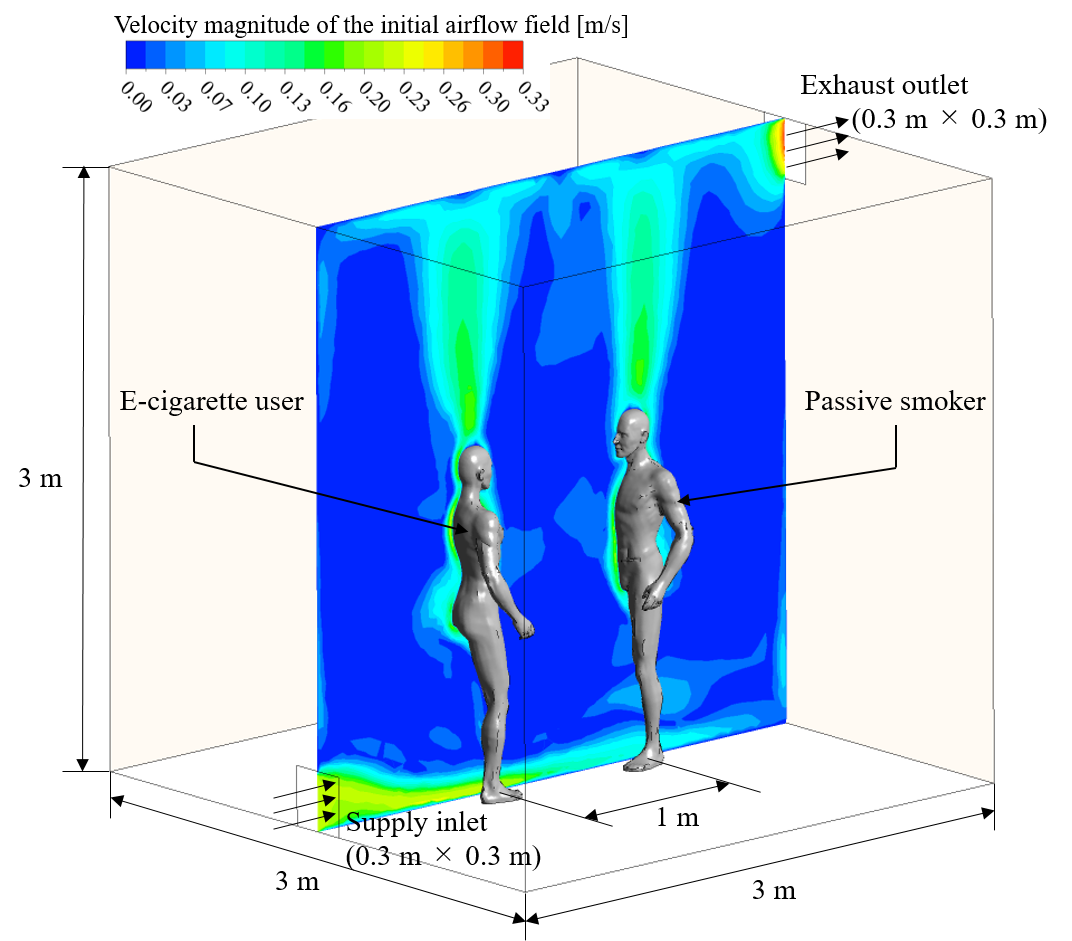


Fig. S3. Analytical domain for the exhaled cannabis dispersion and passive smoking


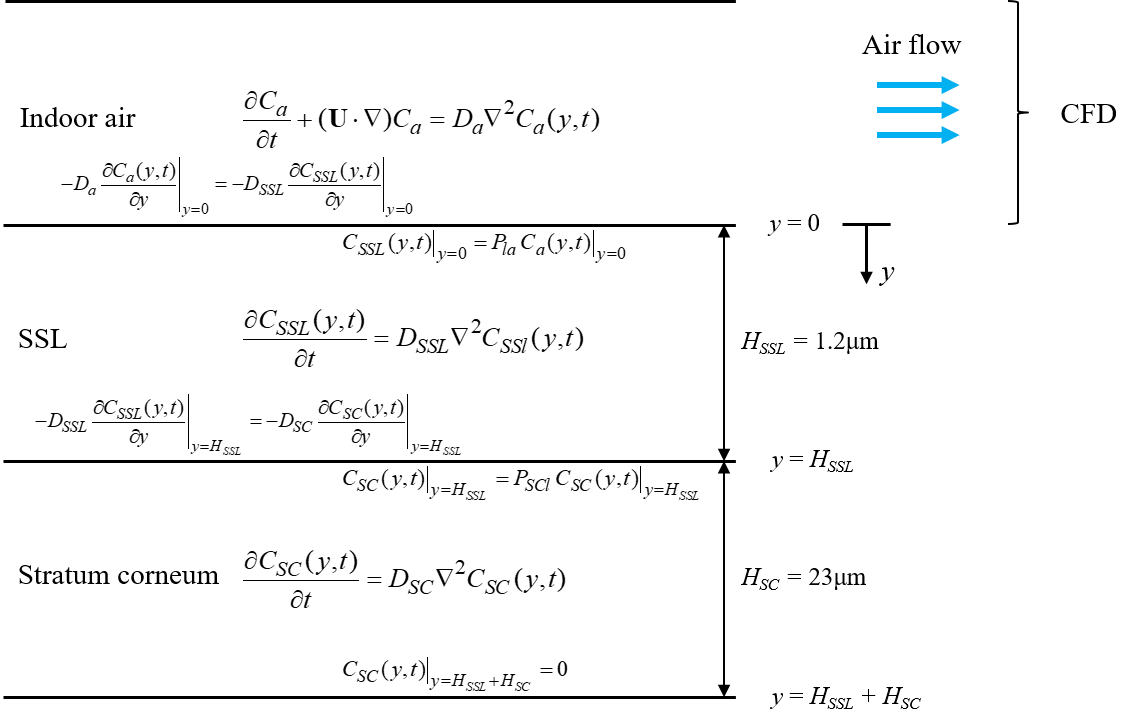


Fig. S4. System of one-dimensional transient diffusion equations for THC dermal absorption

**SI References**

1. K. Kuga, K. Ito, S. J. Yoo, W. Chen, P. Wang, *et al*. First-and second-hand smoke dispersion analysis from e-cigarettes using a computer-simulated person with a respiratory tract model. *Indoor Built Environ*. **27**, 898-916 (2018).
2. J. Xi, P. W. Longest, Effects of Oral Airway Geometry Characteristics on the Diffusional Deposition of Inhaled Nanoparticles, *J Biomech Eng.*, **130**, 011008 (2008).
3. N. L. Phuong, K. Ito, Investigation of flow pattern in a realistic replica model of human respiratory tract using PIV. Build. Environ. **94**, 504-515 (2015).
4. N. L. Phuong, M. Yamashita, S. J. Yoo, K. Ito, Prediction of convective heat transfer coefficient of human upper and lower airway surfaces in steady and unsteady breathing conditions. *Build. Environ.* **100**, 172-185 (2016).
5. J. K. Gupta, C. H. Lin, Q. Chen, Characterizing exhaled airflow from breathing and talking. *Indoor air*. **20**: 31-39 (2010).
6. K. Abe, T. Kondoh, Y. Nagano, A new turbulence model for predicting fluid flow and heat transfer in separating and reattaching flows—I. Flow field calculations. *Int. J. Heat Mass Transfer*. **37**, 139-151 (1994).
7. K. Abe, T. Kondoh, Y. Nagano, A new turbulence model for predicting fluid flow and heat transfer in separating and reattaching flows—II. Thermal field calculations. *Int. J. Heat Mass Transfer*. **38**: 1467-1481 (1995).
8. A. R. Vansickel, J. S. Edmiston, Q. Liang, C. Duhon, C. Connell, et al., Characterization of puff topography of a prototype electronic cigarette in adult exclusive cigarette smokers and adult exclusive electronic cigarette users. *Regul. Toxicol. Pharm*. **98**, 250-256 (2018).
9. K. E. Farsalinos, G. Romagna, D. Tsiapras, S. Kyrzopoulos, V. Voudris, Evaluation of electronic cigarette use (vaping) topography and estimation of liquid consumption: implications for research protocol standards definition and for public health authorities’ regulation. *Int. J. Environ. Res. Public Health.* **10**: 2500-2514 (2013).
10. F. K. Charles, G. R. Krautter, D. C. Mariner, Post-puff respiration measures on smokers of different tar yield cigarettes. *Inhalation toxicol*. **21**, 712-718 (2009).
11. P. Wang, W. Chen, J. Liao, T. Matsuo, K. Ito, *et al*. A device-independent evaluation of carbonyl emissions from heated electronic cigarette solvents. PloS one. **12**, e0169811 (2017).
12. W. Chen, P. Wang, K. Ito, J. Fowles, D. Shusterman, *et al*. Measurement of heating coil temperature for e-cigarettes with a “top-coil” clearomizer. PloS one. **13**, e0195925 (2018).
13. G. Tian, P. W. Longest, Development of a CFD boundary condition to model transient vapor absorption in the respiratory airways. *J. Biomech. Eng.* **132**, (2010).
14. G. Tian, P. W. Longest, Transient Absorption of Inhaled Vapors into a Multilayer Mucus-Tissue-Blood System. *Ann. Biomed. Eng.* **38**, 517 (2010).
15. G. Tian, P. W. Longest, Application of a new dosimetry program TAOCS to assess transient vapour absorption in the upper airways. *Inhalation Toxicol*. **22**, 1047-1063 (2010).
16. Y. Ha, J. H. Kwon, Determination of 1-octanol-air partition coefficient using gaseous diffusion in the air boundary layer. *Environ. Sci. Technol.* **44**, 3041-3046 (2010).
17. M. T. Lopez-Vidriero, Airway secretions: source, biochemical and rheological properties. Lung Biology in Health and Disease, Vol. 5, Respiratory Defence Mechanism. 239-356 (1984).
18. S. Mitragotri, M. E. Johnson, D. Blankschtein, R. Langer, An analysis of the size selectivity of solute partitioning, diffusion, and permeation across lipid bilayers. *Biophys. J.* **77**, 1268-1283 (1999).
19. S. C. George, A. L. Babb, M. E. Deffebach, M. P. Hlastala, Diffusion of nonelectrolytes in the canine trachea: effect of tight junction. *J. Appl. Physiol.* 80, 1687-1695 (1996).
20. R. Sander, Compilation of Henry's law constants (version 4.0) for water as solvent. Atmos. Chem. Phys. 15 (2015).
21. J. C. Anderson, A. L. Babb, M. P. Hlastala, Modeling soluble gas exchange in the airways and alveoli. *Ann. Biomed. Eng.* **31**, 1402-1422 (2003).
22. I. H. Young, P. D. Wagner, Solubility of inert gases in homogenates of canine lung tissue. *J. Appl. Physiol.*  **46**, 1207-1210 (1979).
23. H. E. Buist, L. de Wit-Bos, T. Bouwman, W. H. Vaes, Predicting blood: air partition coefficients using basic physicochemical properties. *Regul. Toxicol. Pharm.* **62**, 23-28 (2012).
24. United Nations Office on Drugs, & Crime, Recommended Methods for the Identification and Analysis of Cannabis and Cannabis Products: Manual for Use by National Drug Testing Laboratories. United Nations Publications, (2009).
25. T. M. Lovestead, T. J. Bruno, Determination of cannabinoid vapor pressures to aid in vapor phase detection of intoxication. *Forensic Chem.* **5**, 79-85 (2017).
26. K. Ito, Toward the development of an in silico human model for indoor environmental design. Proceedings of the Japan Academy, Series B. **92**: 185-203 (2016).
27. S. J. Yoo, K. Ito, Assessment of transient inhalation exposure using in silico human model integrated with PBPK-CFD hybrid analysis. *Sustain. Cities. Soc.* **40**, 317-325 (2018).
28. S. J. Yoo, K. Ito, Numerical prediction of tissue dosimetry in respiratory tract using computer simulated person integrated with physiologically based pharmacokinetic–computational fluid dynamics hybrid analysis. *Indoor Built Environ.* **27**, 877-889 (2018).
29. S. J. Yoo, K. Ito, Multi-stage optimization of local environmental quality by comprehensive computer simulated person as a sensor for HVAC control. *Adv. Build. Energy Res.* 1-18 (2019).
30. C. Wang, S. J. Yoo, K. Ito, Does detailed hygrothermal transport analysis in respiratory tract affect skin surface temperature distributions by thermoregulation model?. *Adv. Build. Energy Res.* 1-21 (2019).
31. P. O. Fanger, Thermal comfort. Analysis and applications in environmental engineering. (1970).
32. S. I. Tanabe, E. A. Arens, F. Bauman, H. Zhang, T. Madsen, Evaluating thermal environments by using a thermal manikin with controlled skin surface temperature. *Ashrae Transactions*, **100**, 39-48 (1994).
33. G. C. Morrison, C. J. Weschler, G. Bekö, Dermal uptake directly from air under transient conditions: advances in modeling and comparisons with experimental results for human subjects. *Indoor air*. **26**, 913-924 (2016).
34. L. Chen, G. Lian, L. Han, Use of “bricks and mortar” model to predict transdermal permeation: model development and initial validation. *Ind. Eng. Chem. Res.* **47**, 6465-6472 (2008).
35. S. Mitragotri, A theoretical analysis of permeation of small hydrophobic solutes across the stratum corneum based on scaled particle theory. *J. Pharm. Sci.* **91**, 744-752 (2002).
36. J. Kushner IV, W. Deen, D. Blankschtein, R. Langer, First‐principles, structure‐based transdermal transport model to evaluate lipid partition and diffusion coefficients of hydrophobic permeants solely from stratum corneum permeation experiments. *J. Pharm. Sci.* **96**, 3236-3251 (2007).
37. J. M. Nitsche, T. F. Wang, G. B. Kasting, A two-phase analysis of solute partitioning into the stratum corneum. *J. Pharm. Sci.* **95**, 649-666 (2006).
