# Supplementary figures and images for: Secondary indoor air pollution and passive smoking associated with cannabis smoking using electric cigarette device–demonstrative in silico study
Source: PLoS Comput Biol. 2021 May 13;17(5):e1009004. doi: 10.1371/journal.pcbi.1009004 (PMC8148323; doi:10.1371/journal.pcbi.1009004)

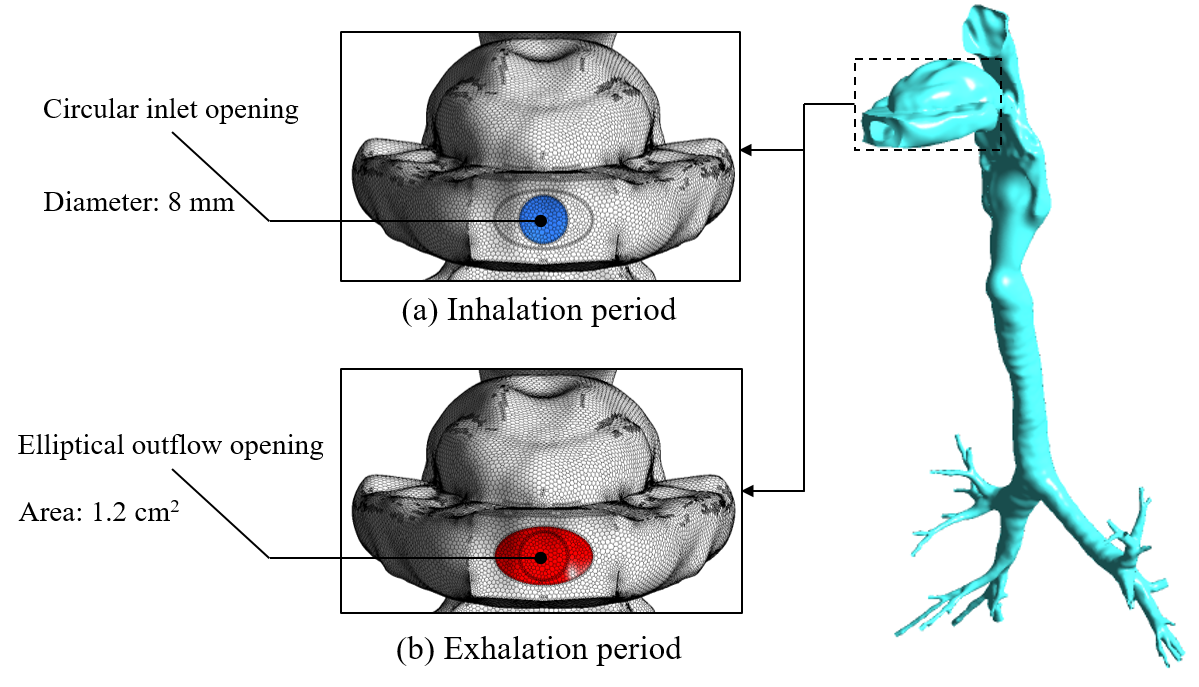

Supplement: S1 Fig — (TIF) [file pcbi.1009004.s002.tif]

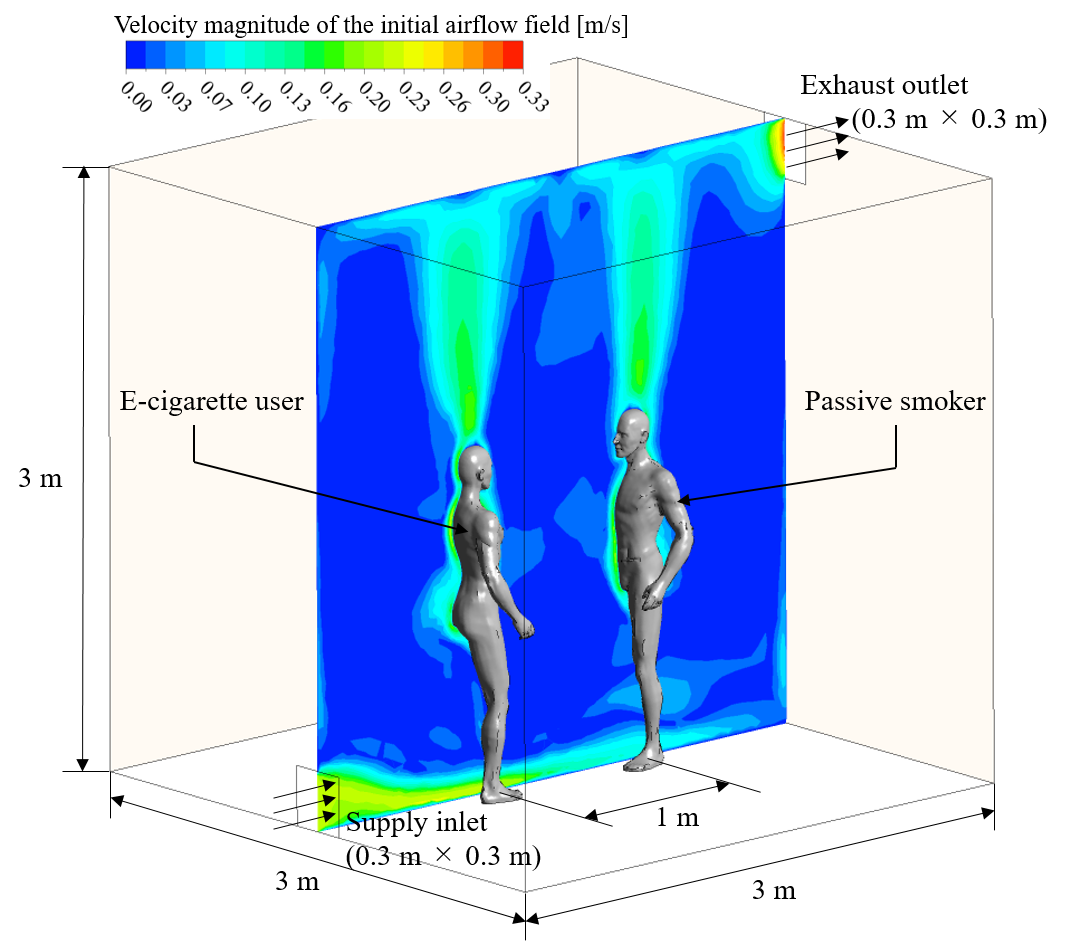

Supplement: S3 Fig — (TIF) [file pcbi.1009004.s004.tif]
